# TFE3 Translocation-Associated Renal Cell Carcinoma Presenting as Avascular Necrosis of the Femur in a 19-Year-Old Patient: Case Report and Review of the Literature

**DOI:** 10.1155/2011/432917

**Published:** 2011-10-05

**Authors:** T. Nelius, I. Al-Khalil, J. Vordermark, K. Rinard-Holden, T. Cammack, V. Mamlok, S. Filleur

**Affiliations:** ^1^Department of Urology, Texas Tech University Health Sciences Center, Medical Office Plaza, Lubbock, TX 79415, USA; ^2^Department of Pediatrics, Texas Tech University Health Sciences Center, Lubbock, TX 79430-8115, USA; ^3^Department of Pathology, Texas Tech University Health Sciences Center, Lubbock, TX 79430-9406, USA

## Abstract

In the United States, renal cell carcinoma (RCC) accounts for approximately 3% of adult malignancies and 90–95% of all neoplasms arising from the kidney. According to the National Cancer Institute, 58 240 new cases and 13 040 deaths from renal cancer will occur in 2010. RCC usually occurs in older adults between the ages of 50 and 70 and is rare in young adults and children. We describe a case of a TFE3 translocation-associated RCC in a 19-year-old patient presenting as avascular necrosis of the femur. Due to the rarity of this malignancy, we present this case including a review of the existing literature relative to diagnosis and treatment.

## 1. Introduction

In the United States, renal cell carcinoma (RCC) accounts for approximately 3% of adult malignancies and 90–95% of all neoplasms arising from the kidney. The incidence varies depending on racial and ethnic characteristic [1]. According to the National Cancer Institute an estimated 58 240 new cases and 13, 040 deaths from renal cancer will occur in 2010. RCC usually occurs in older adults between the ages of 50 and 70 and is rare in young adults and children [2]. Predisposing conditions, known to increase the risk of RCC, include cigarette smoking, obesity, hypertension, and diabetes mellitus. Several studies suggest also an association between development of RCC and other factors, such as physical activity, alcohol consumption, acrylamide intake, occupational and environmental exposure to trichloroethylene and heavy metals such as cadmium, lead, and arsenic, and parity in women [1]. Genetic susceptibility was also shown to play a major role in inherited RCC, for example, Hippel-Lindau (VHL) disease [3], shorter telomere length in peripheral blood lymphocyte DNA [4]. Additionally, multiple other genetic variations were found to be associated with RCC risk; however only limited evidence is available [4–12]. Nephroblastoma are Wilm's tumor are the most common types of kidney cancer in children and younger adults. It comprises approximately 1.2% of all kidney cancers [1]. The clear cell subtype of RCC is most common, followed by RCC not otherwise specified, papillary, and chromophobe subtypes [1]. The different histological subtypes have well-documented clinical and genetic characteristics [13, 14]. The first detailed morphological characterization of these tumors was published by Argani et al. in 2001 [15]. In 2004, the Xp11 translocation RCC was introduced as a genetically distinct entity into the World Health Organization classification of renal neoplasms [16, 17]. This subtype occurs especially in the pediatric age group, where it accounts for at least one-third of RCCs and for 15% of RCCs in patients <45 years of age [18]. Most of these papillary RCCs exhibit certain cytogenetic abnormalities, including t(X; 1)(p11.2; q21), t(X; 1)(p11.2; p34), (X; 17)(p11.2; q25.3), and inv(X)(p11.2; q12) [19]. These translocations result in gene fusions involving the TFE3 transcription factor gene which maps to this locus [20–23]. Even though the functions of TFE3 are not completely defined yet, it has been described as being important for stimulation of the plasminogen activator inhibitor 1 (PAI-1) gene promoter by TGF-b in conjunction with Smad3 and Smad4 [24] and for osteoclast development [25]. The diagnosis of an Xp11 translocation can be made by immunohistochemistry with antibodies against TFE3. TFE3 is not detected by this method in normal tissue. 

Information about the natural history is sparse; however the evidence is mounting that patients with metastatic Xp11 translocation RCC have aggressive disease that usually presents at an advanced stage [18, 26–32]. Herein, we describe a case of a TFE3 translocation-associated RCC in a 19-year-old patient presenting initially as avascular necrosis of the femur. Due to the rarity of this malignancy, we present this case including a review of the existing literature relative to diagnosis and treatment. We will also characterize the tumor by immunohistochemistry and its response to different treatment regimens. By documenting the response to various treatments this paper should help to find optimal treatment regimens for this particular clinical situation.

## 2. Case Report

### 2.1. Initial Presentation

**Figure 1:**
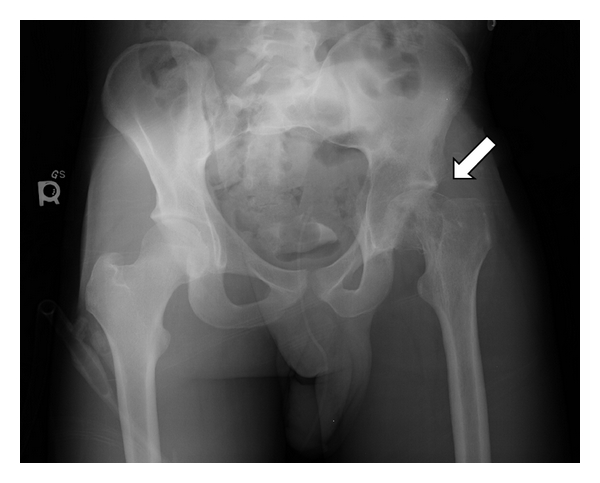
X-ray of the pelvis (anterior-posterior) showing pathological fracture of the left femur neck (arrow).

Our patient was a 19-year-old male who had approximately one year of mild-to-moderate low back pain, for which he was being treated by a chiropractor. After development of left hip pain, X-ray examination showed osteopenia of the left femoral head and neck. The diagnosis of Perthes' disease was made and treated accordingly. The patient was placed on nonweight-bearing status of the left hip after a fall. 3 months later he suffered a pathological fracture to the left femur neck (Figure 1). A CT scan of the abdomen and pelvis revealed a large left-sided renal mass measuring 11.5 × 10.7 cm, consistent with a renal neoplasm. The patient was referred to our institution for management.

### 2.2. Hospital Course

The patient had no relevant past medical and surgical history. Family history was noncontributory, specifically no history of malignant diseases. Social history was also unremarkable, particularly no history of tobacco, alcohol, or drug use or exposure to toxins or carcinogens. Physical examination revealed a normally developed 19-year-old male. Tenderness of the left hip region on deep palpation was encountered, as well as tenderness to palpation in the lower back. A palpable firm mass was encountered in the left-sided abdomen. 

Admission laboratory studies were WBC 7.7 kU/L, HGB 13.1 g/dL, RBC 4.01 M/UL, platelets 241 kU/L, and hematocrit 37.7%. His blood urea nitrogen was 11 mg/dL, creatinine 0.7 mg/dL. Urinalysis was positive for nitrite, blood, and leucocytes. Liver function test was ALT 10 intl units/L, AST 54 intl units/L, LD 327 units/L, total bilirubin 0.4 mg/dL, total protein 6.9 g/dL, albumin 3.9 g/dL. Alkaline phosphatase was 79 intl units/L.

**Figure 2:**
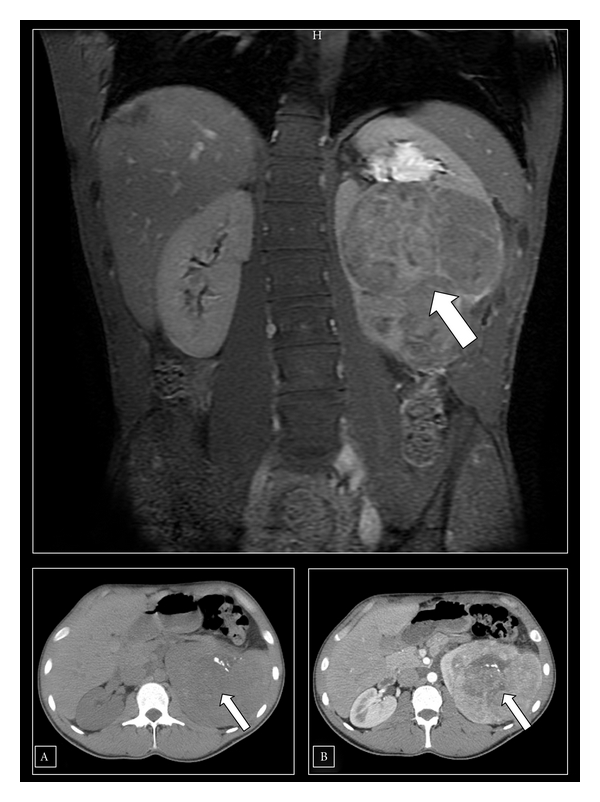
MRI and CT scan (inset (a) without IV contrast and (b) with IV contrast) of the abdomen and pelvis showing a large heterogeneously enhancing (inset (b)) exophytic left renal mass measuring 11.6 × 10.7 cm with multiple small calcifications (arrow inset (a)).

Initial differential diagnosis included possible metastatic renal cell cancer, Wilm's tumor, or lymphoma. CT scan of the abdomen and pelvis showed a large heterogeneously enhancing exophytic left renal mass measuring 11.6 × 10.7 cm, infiltrating the renal pelvis and lower pole calyceal system. The mass had multiple small calcifications. The mass extended to the left lateral and posterior abdominal wall without evidence of infiltration or solid organ involvement and without significant lymphadenopathy. The findings could be confirmed by MRI (Figure 2). CT scan examination of the head, lung, and mediastinum was normal. 

There was an osteolytic lesion involving the L4 vertebral body anteriorly with prevertebral soft tissue component and osteolytic lesions involving the left proximal femur and left acetabulum.

**Figure 3:**
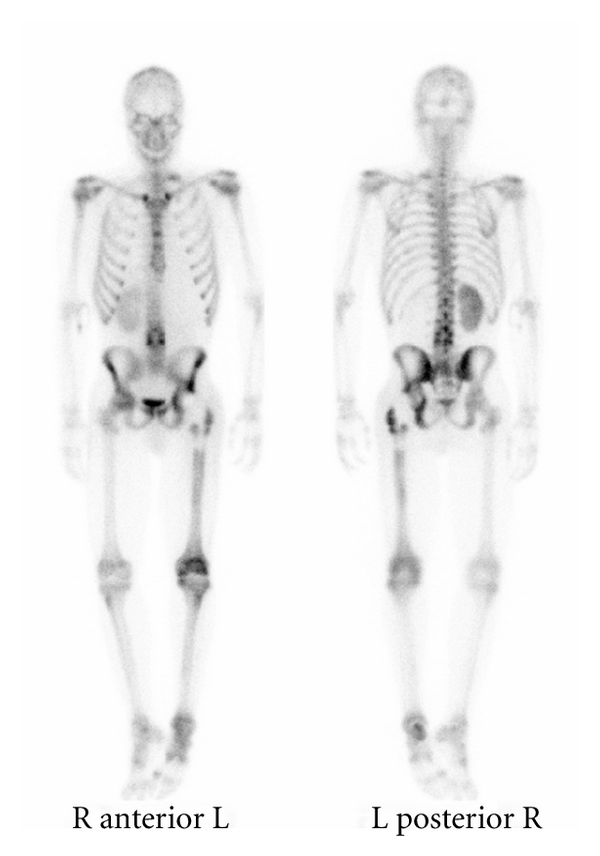
Whole body bone scan performed after left hip replacement showing mild salt-and-pepper appearance to the skull, sternum, and posterior ribs. The axial skeleton and the posterior left hemipelvis showed increased radionuclide activity suggestive of metastatic disease to these regions.

The bone scan demonstrated a very mild salt-and-pepper appearance to the skull, sternum, and posterior ribs. The axial skeleton showed increased radionuclide activity throughout the entire diaphysis of the left femur as well as the lower lumbar spine and midsternum. There was also increased radionuclide activity at the posterior left hemipelvis, suggestive of metastatic disease to the axial skeleton and the lower appendicular skeleton (Figure 3).

**Figure 4:**
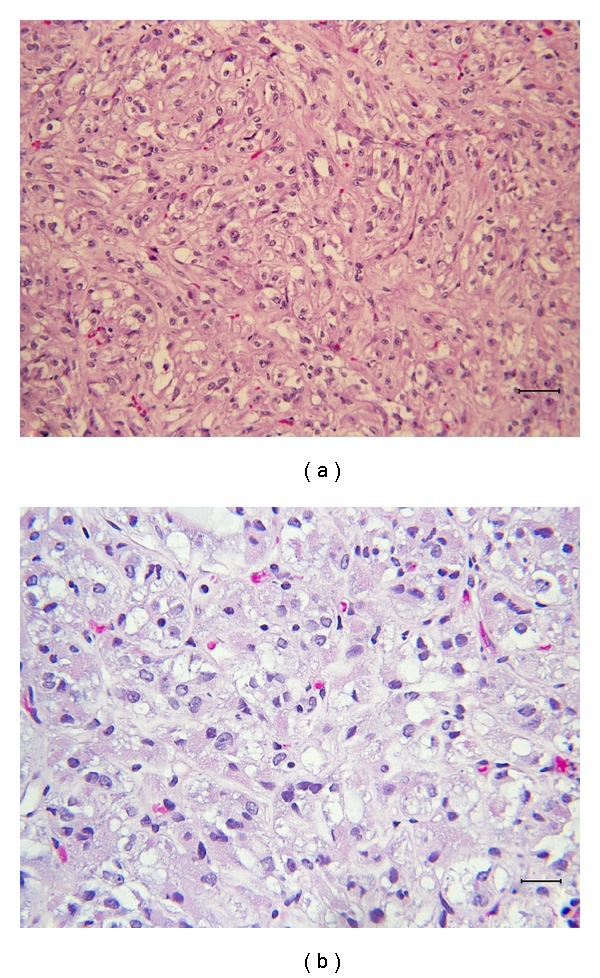
H&E stainings ((a) 200x and (b) 400x magnification) of the tumor showing a mixture of spindle cell (predominant) and epithelioid cells with abundant amphophilic, clear, and focally granular pink cytoplasm. Epithelioid cells often line spaces, spindle cells form fascicles.

Percutaneous biopsies of the tumor showed a mixture of spindle cell (predominant) and epithelioid cells with abundant amphophilic, clear, and focally granular pink cytoplasm. Epithelioid cells often line spaces, spindle cells form fascicles. Necrosis is not present (Figure 4). The selective immunophenotype was inconclusive. Immunoperoxidase stains: are as following. Positive: MS Actin (focally positive, 0-1+); Myo-D1 (Clarient); EMA (rare); CD10 (only RARE positive cells). Negative: Vimentin (background vessels positive, no clearly positive tumor cells), CKAE1/AE3, Desmin (some vessels positive); Inhibin, RCC, Melan A(MART1)(Clarient). A variety of tumors were in the differential diagnosis included, including alveolar soft part sarcoma, alveolar rhabdomyosarcoma, chromophobe carcinoma, translocation renal cell carcinoma, and atypical angiomyolipoma, but the mixed pattern of histology required further immunohistochemistry for final diagnosis. 

The patient was discussed at a multidisciplinary tumor board meeting, where it was agreed that a radical tumor nephrectomy with the intention of tumor debulking was indicated. The patient was also scheduled for curettage and bone grafting of the metastatic femur lesion and left periacetabular region and calcar-replacing left total hip arthroplasty. On hospital days 5 and 10 the patient underwent left radical nephrectomy via chevron incision and orthopedic surgery.

 Intraoperatively the retroperitoneum demonstrated extensive number of dilated blood vessels and dramatically dilated lymphatics, measuring up to 6–8 mm in diameter coursing over the surface of the kidney, Gerota's fascia, and the mesentery to the left colon. These dilated lymphatics extended over the aorta and up to the renal hilum. Both surgeries went uneventful. 

**Figure 5:**
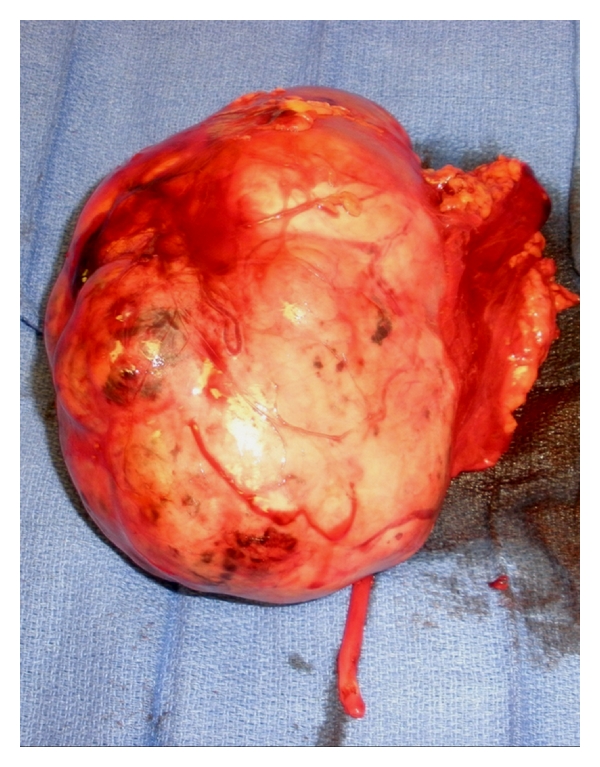
Radical nephrectomy specimen demonstrating an intact kidney mass without perinephric fat measuring 18 × 12 × 7 cm and weight of 920 g. The tumor appears as solid, tan-pink, and well-circumscribed mass grossly confined by the renal capsule. Of note is the extensive number of dilated blood vessels and lymphatics.

Pathological examination showed an intact kidney without perinephric fat measuring 18 × 12 × 7 cm and weight of 920 g. Approximately 90% of the kidney had been replaced by the tumor, except for a rim of upper pole that measured 6 × 3 × 2 cm. The tumor was unifocal measuring 12 cm greatest dimension. The tumor was a solid, tan-pink mass with hemorrhage and necrosis, limited to the kidney, well circumscribed, and grossly confined by the renal capsule. The uninvolved renal parenchyma was unremarkable (Figure 5). 

Microscopically, the renal tumor was composed of relatively large and uniform epithelioid cells with clear to vacuolated to eosinophilic cytoplasms. The tumor showed a nesting or trabecular growth patterns. The nuclei show intermediate grade features but overall have a bland appearance. Mitotic activity was sparse. 

**Figure 6:**
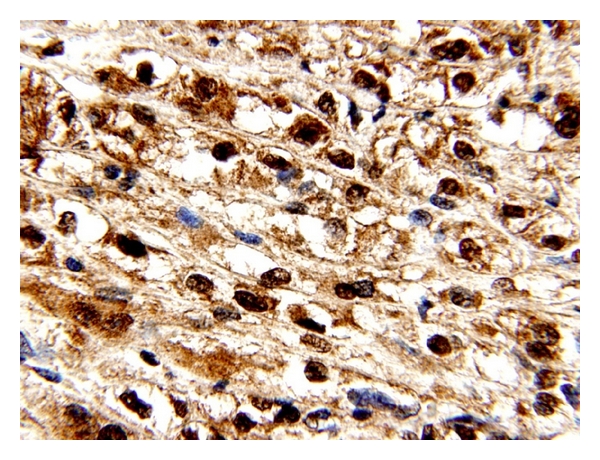
Immunostaining demonstrating strong nuclear positivity for TFE3 protein with only minimal background staining.

The immunostainings were negative for melan-A and the melanoma cocktail. This tumor was also nonreactive for EMA, CD10, pancytokeratin, CK7, CD68, CD34 and E-cadherin Mart 1, HMB45, and CKAE1/AE3. Vimentin highlighted an occasional tumor cell. PAS with and without diastase and colloidal iron stains was equivocal (Figure 6). After reviewing the immunostains and given the fact that the tumor is clearly metastatic to the young man's femur, we included alveolar soft part sarcoma in the differential diagnosis. We performed further immnostains which confirm that the tumor is negative for pancytokeratin and melan-A. This tumor displayed focal positivity for smooth muscle actin and vimentin. Finally, nuclear positivity for TFE-3 was present (Figure 6). TFE-3 expression would imply that there is an Xp11.2 translocation. These findings were consistent with classification of the tumor as a TFE3 translocation-associated renal cell carcinoma. This is supported by the strong nuclear positivity for TFE3 in the absence of significant background staining in our hands, the negativity for vimentin (which would be highly unusual for epithelioid angiomyolipoma), as well as the negativity in everyone's hands for all melanocytic markers. The biopsy from the left femur demonstrates a similar histology. The tumor was staged accordingly as Xp11.2 translocation renal cell carcinoma with smooth muscle actin positivity, pT2b, pNx, pM1_(bone) _.

Based on the histopathological characteristics of the tumor (clear cell carcinoma with sarcomatoid features) and its metastatic spread, chemotherapy according to the *AREN0321Treatment Regimen for High Risk Renal Tumors* was initiated. The treatment regimen of a 4-week cycle included Cyclophosphamide: IV over 1 hour 1200 mg/m^2^/day, week 1; Doxorubicin: IV over 120 minutes on Day 1 dose 45 mg/m^2^/day; Vincristine: IV Day 1 wks 1–3 = 1.5 mg/m^2^/day; Carboplatin: IV over 1 hour on Day 1 of week 4; cyclophosphamide: IV over 15–30 minutes on Days 1–4 of week 4 dose 440 mg/m^2^/day. Additionally, the patient received palliative radiation therapy with a total dose of 3500 cGy in 15 fractions using the 3D conformal technique with the 15 MV unit to the pelvis/left hip and to the C-spine and left shoulder with a total dose of 3150 cGy given in 8 fractions also using the 3D conformal technique with the 6 MV unit. The patient developed disease progression under this chemotherapy/radiation regimen and was started on Vincristine: IV Day 1 of weeks 1-2 dose 1.5 mg/m^2^/dose and Irinotecan: IV 20 mg/m^2^/daily X 5 according to the *COG protocol* for unresponsive renal masses. No significant response was noted. It was decided to start the patient on a combination regimen consisting of Sorafenib plus Irinotecan. This combination resulted in stable disease for approximately 8 weeks. However, this combination caused significant diarrhea, and Sorafenib was stopped. Finally, the patient was treated with a combination of Irinotecan, Temsirolimus, and Bevacizumab. Despite a negative PET scan the patient developed clinically progressive disease under treatment and died secondary to multisystem failure. 

## 3. Discussion

Solid tumors of the kidney are the third most common malignancies in children and young adults. While Wilms tumor being the most frequent and well-studied entity, RCC is rare and is responsible for only 0.3% of all malignancies and up to 6.3% of all malignant renal tumors in this age group [33]. Because of this fact little is known regarding possible causes of RCC in this age group and treatment. A few case series reports have been published in the literature [33–35]. Indolfi et al. published in 2003 one of the largest clinicopathological studies involving 41 patients with a median age of 10 years [33]. In this study, clear cell carcinoma had with 59% of the cases a similar incidence rate compared to adult series. In addition, the overall outcome of RCC in pediatric patients is comparable to the outcome in adults. Several studies defined the stage of the disease at presentation as major factor influencing the prognosis. Tumors localized to the kidney have a better prognosis than tumors with regional lymph node involvement or distant metastatic disease [33, 34]. On the contrary, Geller and Dome found that lymph node involvement is not an adverse prognostic factor for pediatric RCCs in contrast to the presence of distant metastases [35]. As described for the adult patient population an obvious association of von Hippel-Lindau (VHL) disease or tuberous sclerosis complex with RCC exists [36, 37]. In case of the VHL, a germ line mutation on chromosome 3p is linked to the development of RCC [38]. It is noteworthy that RCCs in children and young adults often have a pseudopapillary architecture. Over the last 20 years a new cytogenetic subtype involving the chromosome band Xp11.2 in pediatric and adult RCC patients has been described [20–23]. The t(X; 1)(p11; q21) is the most frequent translocation resulting in the PRCC-TFE3 gene fusion [20]. Although TFE3 RCC represents only a relatively small portion of renal tumors in childhood and young adults, these tumors seem to have different characteristics in terms of histologic patterns, biologic behavior, and possible response to treatment. Malouf et al. analyzed the benefit of targeted therapy (vascular endothelial growth factor receptor- (VEGFR-) targeted agents and/or mammalian target of rapamycin (mTOR) inhibitors) in patients with Xp11 translocation/TFE3 fusion gene metastatic RCC [32]. In their retrospective study, patients with Xp11 translocation mRCC displayed aggressive disease with a median progression-free survival (PFS) of 2 months when receiving a cytokine-based regimen and an 11% response rate. VEGFR-targeted and/or mTOR inhibitor treatment demonstrated an objective response rate of 33% of the patients. Interestingly, patients treated with sunitinib had an 8.2-month PFS, which is similar to clear cell RCC. The group concluded that response to targeted therapy does not depend necessarily on RCC subtype [32]. As seen in our patient chemotherapy is not effective. Sunitinib is achieved in combination with irinotecan stable disease. This finding is in concordance with the results form Malouf et al., which described a better PFS in patients on first-line sunitinib than in those receiving cytokines. Sunitinib seems to be effective in patients with TFE3 RCC as 7 of 14 of their treated patients achieved a partial (*n* = 6) or complete (*n* = 1) response [32].

In summary, TFE3 RCC is a rare malignancy where the genetic background may not only contribute to tumorigenesis, but also determine the response to chemotherapy and targeted therapy. Therefore it is necessary to diagnose this tumor entity accurately. Histomorphological and immunohistochemical features were previously described by Argani et al., which may help pathologists in distinguishing these neoplasms from their mimics [20, 39]. Because of the small number of *TFE3 *gene fusion-related renal tumors described in the literature, the exact biologic behavior and impact of current treatment modalities remain to be uncertain. Increased awareness among urologists, pathologists, and oncologist is necessary in order to help in identifying more cases of this phenotype in the future. Prospective randomized studies on novel targeted agents are needed to identify the optimal treatment strategy for this specific patient population. In addition, a better characterization of the genetics of these translocations will help to develop more specific drugs.
